# Publisher Correction: Characterising within-hospital SARS-CoV-2 transmission events using epidemiological and viral genomic data across two pandemic waves

**DOI:** 10.1038/s41467-022-28681-2

**Published:** 2022-02-17

**Authors:** Benjamin B. Lindsey, Ch. Julián Villabona-Arenas, Finlay Campbell, Alexander J. Keeley, Matthew D. Parker, Dhruv R. Shah, Helena Parsons, Peijun Zhang, Nishchay Kakkar, Marta Gallis, Benjamin H. Foulkes, Paige Wolverson, Stavroula F. Louka, Stella Christou, Amy State, Katie Johnson, Mohammad Raza, Sharon Hsu, Thibaut Jombart, Anne Cori, Sheffield COVID-19 Genomics Group, Dhruv R. Shah, Katie Johnson, Sharon Hsu, Thushan I. de Silva, Alison Cope, Nasar Ali, Rasha Raghei, Joe Heffer, Nikki Smith, Max Whiteley, Manoj Pohare, Samantha E. Hansford, Luke R. Green, Dennis Wang, Michael Anckorn, Adrienn Angyal, Rebecca Brown, Hailey Hornsby, Mehmet Yavuz, Danielle C. Groves, Paul J. Parsons, Rachel M. Tucker, Magdalena B. Dabrowska, Thomas Saville, Jose Schutter, Matthew D. Wyles, The COVID-19 Genomics UK (COG-UK) consortium, Cariad Evans, CMMID COVID-19 working group, Nicholas G. Davies, Carl A. B. Pearson, Matthew Quaife, Damien C. Tully, Sam Abbott, Cariad M. Evans, David G. Partridge, Katherine E. Atkins, Stéphane Hué, Thushan I. de Silva

**Affiliations:** 1 The Florey Institute for Host-Pathogen Interactions & Department of Infection, Immunity and Cardiovascular Disease, Medical School, University of Sheffield, Sheffield, UK; 2 Sheffield Teaching Hospitals NHS Foundation Trust, Sheffield, UK; 3 Centre for Mathematical Modelling of Infectious Diseases, London School of Hygiene and Tropical Medicine, London, UK; 4 Department of Infectious Disease Epidemiology, London School of Hygiene and Tropical Medicine, London, UK; 5 Health Emergencies Programme, World Health Organization, Geneva, Switzerland; 6 Sheffield Biomedical Research Centre, The University of Sheffield, Sheffield, UK; 7 Sheffield Bioinformatics Core, The University of Sheffield, Sheffield, UK; 8 The Department of Neuroscience/Neuroscience Institute, The University of Sheffield, Sheffield, UK; 9 MRC Centre for Global Infectious Disease Analysis, Department of Infectious Disease Epidemiology, School of Public Health, Imperial College London, London, UK; 10 Usher Institute, The University of Edinburgh, Edinburgh, UK; 11 MRC Unit The Gambia at the London School of Hygiene and Tropical Medicine, Fajara, The Gambia; 12 IT services, The University of Sheffield, Sheffield, UK; 13 Department of Computer Science, The University of Sheffield, Sheffield, UK; 14 Department of Animal and Plant Sciences, The University of Sheffield, Sheffield, UK

**Keywords:** Epidemiology, SARS-CoV-2, Genomics

Correction to: *Nature Communications* 10.1038/s41467-022-28291-y, published online 3 February 2022.

The original version of this Article contained errors in the Abstract, main text, Table 1, and References.

An apostrophe was included in the Abstract “25th J’uly 2020”, which has been corrected to “25th July 2020”.

In the main text and References, the phrase “Identifying within-hospitalSARS-CoV-2 transmission” was missing a space between “hospital” and “SARS-CoV-2”, which has been corrected to “Identifying within-hospital SARS-CoV-2 transmission”.

In the main text, there were two uses of the phrase “hospital-acquiredSARS-CoV-2 infections” which were also missing a space between “acquired” and “SARS-CoV-2”, which have been corrected to “hospital-acquired SARS-CoV-2 infections”.

In Table 1, there was an additional space in “Community onset— community associated”, which has been corrected to “Community onset—community associated”.

These corrections have been made in both the PDF and HTML versions of the Article.

